# Resistance to visceral obesity is associated with increased locomotion in mice expressing an endothelial cell‐specific fibroblast growth factor 1 transgene

**DOI:** 10.14814/phy2.14034

**Published:** 2019-04-10

**Authors:** Tyler Keeley, Aleksandr Kirov, Woon Yuen Koh, Victoria Demambro, Ivy Bergquist, Jessica Cotter, Peter Caradonna, Matthew E. Siviski, Bradley Best, Terry Henderson, Clifford J. Rosen, Lucy Liaw, Igor Prudovsky, Deena J. Small

**Affiliations:** ^1^ Department of Chemistry and Physics College of Arts and Sciences University of New England Biddeford Maine; ^2^ Center for Molecular Medicine Maine Medical Center Research Institute Scarborough Maine; ^3^ Department of Mathematical Sciences College of Arts and Sciences University of New England Biddeford Maine; ^4^ Center for Excellence in Neuroscience College of Medicine University of New England Biddeford Maine

**Keywords:** Adipogenesis, adiposity, FGF1, glucose tolerance, locomotor activity, metabolism

## Abstract

Overdevelopment of visceral adipose is positively correlated with the etiology of obesity‐associated pathologies including cardiovascular disease and insulin resistance. However, identification of genetic, molecular, and physiological factors regulating adipose development and function in response to nutritional stress is incomplete. Fibroblast Growth Factor 1 (FGF1) is a cytokine expressed and released by both adipocytes and endothelial cells under hypoxia, thermal, and oxidative stress. Expression of Fibroblast Growth Factor 1 (FGF1) in adipose is required for normal depot development and remodeling. Loss of FGF1 leads to deleterious changes in adipose morphology, metabolism, and insulin resistance. Conversely, diabetic and obese mice injected with recombinant FGF1 display improvements in insulin sensitivity and a reduction in adiposity. We report in this novel, in vivo study that transgenic mice expressing an endothelial‐specific FGF1 transgene (FGF1‐Tek) are resistant to high‐fat diet‐induced abdominal adipose accretion and are more glucose‐tolerant than wild‐type control animals. Metabolic chamber analyses indicate that suppression of the development of visceral adiposity and insulin resistance was not associated with alterations in appetite or resting metabolic rate in the FGF1‐Tek strain. Instead, FGF1‐Tek mice display increased locomotor activity that likely promotes the utilization of dietary fatty acids before they can accumulate in adipose and liver. This study provides insight into the impact that genetic differences dictating the production of FGF1 has on the risk for developing obesity‐related metabolic disease in response to nutritional stress.

## Introduction

Although diets high in carbohydrates and/or saturated fat often result in the development of obesity and obesity‐related pathologies, patterns of adipose accretion, and incidence of metabolic disease differ among individuals with similar dietary and exercise regimens (Bouret et al. 2015). Sex and other genetic factors impact an individual's molecular, cellular, and physiological response to nutritional stress caused by overnutrition. For example, transcriptome analyses have identified a variety of genes encoding metabolic hormones as well as proteins associated with adipogenesis, angiogenesis and inflammation that are differentially regulated by not only diet, but also by sex and disease (Urs et al. 2004; Koza et al. 2006; Hagobian et al. 2009; Viguerie et al. 2012). However, despite these gains in knowledge regarding genetic risks factors associated with obesity and metabolic disease susceptibility, the identification of “obese” genes and/or their functional significance in metabolic health is still incomplete.

One genetic factor likely to impact adiposity and energy homeostasis is the potent cytokine Fibroblast Growth Factor 1 (FGF1). FGF1 is one of 18 ligands that bind to four differentially spliced receptors in mammals (Ornitz and Itoh 2015; Yang et al. 2015). FGF receptors (FGFRs) are ubiquitously expressed tyrosine kinases that activate downstream effectors including Src, MAPK(Erk1/2), PLC*γ* and the transcriptional regulators Fos/Jun(Ap1) (Friesel and Maciag 1995; Ornitz and Itoh 2015). FGF1 has the potential to affect cell fate decisions in every cell type as it can bind to all FGFRs and their splice variants (Stauber et al. 2000; Zhang et al. 2006; Beenken et al. 2012; Nguyen et al. 2014). FGF1 gene transcription is highly regulated and results in the production of three different mRNA species, each arising from unique transcription start sites in the *fgf1* promoter (Alam et al. 1996; Madiai et al. 1999; Zhang et al. 2001). Unlike most other FGF family members (with the exception of FGF2), the release of FGF1 into the extracellular environment is also tightly regulated through a stress‐induced, nonclassical secretory pathway requiring formation of a copper‐dependent complex that facilitates the transport of FGF1 from the cytoplasm through the plasma membrane (Prudovsky et al. 2008). The role of FGF1 as a regulator of adipogenesis has been explored over the last 25 years in human and murine cell culture systems, although whether it stimulates or represses adipocyte differentiation is dependent on cell type and exposure period (Hutley et al. 2004, 2011; Widberg et al. 2009). FGF1 expression in adipose is elevated in obese humans (Gabrielsson et al. 2002; Mejhert et al. 2010; Gerhard et al. 2014) and in rodents administered a high‐fat diet (Koza et al. 2006; Jonker et al. 2012). Mice lacking a functional *fgf1* gene(fgf1^−/−^) display defects in adipose remodeling and vascularization, insulin resistance, and steatosis in response to diets high in saturated fats (Jonker et al. 2012). Conversely, both parenteral and intracerebroventricular administration of recombinant FGF1 improves insulin sensitivity and lipid metabolism in diabetic mice, further supporting a role for FGF1 as a regulator of metabolic homeostasis (Suh et al. 2014; Scarlett et al. 2016). However, the ability of transgenically expressed FGF1 to prevent the deleterious effects of excess intake of saturated fats on adipose development, glucose homeostasis, and lipid metabolism in vivo has yet to be reported.

We conducted a novel, FGF1 gain‐of‐function study using our double transgenic, endothelial‐specific FGF1‐Tek murine model (Kirov et al. 2012) to test the hypothesis that FGF1 protects against high‐fat diet (HFD)‐induced visceral adipose accretion, glucose intolerance, and liver steatosis. Transgenic expression of FGF1 promoted a lean phenotype with enhanced glucose tolerance and decreased serum lipids. FGF1‐Tek mice were also more active than FVB controls – a behavioral trait that likely contributed to their resistance to obesity. Interestingly, transgenic FGF1 also altered adipose morphology, the adipogenic potential of preadipocytes and increased the expression of brown adipose markers in the visceral white adipose depot. The data presented in this report underscore the importance of FGF1 as a modulator of adipose biology and energy homeostasis and supports its capacity as an antiobesogenic agent.

## Materials and Methods

### Animals and high‐fat diet protocols

In this study, we used FGF1‐Tek mice with endothelial‐specific FGF1 expression. The FGF1‐Tek mouse strain containing the exogenous *fgf1* gene under control of the TRE‐Tight promoter and rtTA under the endothelial *tek* promoter was produced as described in the publication by Kirov et al. (2012). Wild‐type FVB/N mice served as a control (FVB Control) as the mice were produced and maintained in this strain. Five‐week old, female FVB Control and FGF1‐Tek mice (Kirov et al. 2012) were fed a high‐fat diet (45%, Research Diets, Inc.), ad libitum until puberty (4‐week study) or into adulthood (14‐week study). To enhance endothelial cell expression of the FGF1 transgene, 0.66 mg/mL doxycycline was added to drinking water supplemented with 1% sucrose to increase palatability 48 h before the dietary regimen was initiated and continued until euthanasia. To control for any effects of the antibiotic, both FVB Control and FGF1‐Tek strains received doxycycline. Mice were fasted for 6–8 h prior to euthanasia by CO_2_ inhalation. This study was approved by the Institutional Animal Care and Use Committees of The University of New England (approval ID# UNE‐20140203SMALD) and Maine Medical Center (approval ID# 0701).

### Determination of adiposity

Longitudinal body composition of anesthetized mice was obtained using PIXImus dual energy X‐ray absorptiometry (GE‐Lunar, Madison, WI, USA). Mice were placed ventral side down with each limb and tail positioned away from the body. X‐ray absorptiometry data were gathered and processed with manufacturer‐supplied software (Lunar PIXImus 2,vers. 2.1).

### Tomography

The torso of each anesthetized mouse was scanned at an isotropic voxel size of 76 microns (45 kV, 177 *μ*A, 300 msec integration time) with a vivaCT 40 scanner (Scanco Medical Inc.). Two‐dimensional gray‐scale image slices were reconstructed between the proximal end of L1 and the distal end of L5 into a three‐dimensional tomography. The region of interest (ROI) for each animal was defined based on skeletal landmarks from the gray‐scale images.

### Metabolic chamber assays

Female FVB Control and FGF1‐Tek Mice were placed individually into Promethian Metabolic Chambers (Sable Systems International) for 5 days under standard 12 h light/dark cycle to obtain continuous measurements of parameters described in figure legends. Measurements were taken 24 h/day during a 72‐h timeframe following an initial 24 h acclimation period. Measurement acquisition was obtained as described (Demambro et al. 2015). Statistical evaluation of the data is described below and within figure legends.

### Glucose tolerance tests

Prior to the start of the metabolic chamber assays, female FVB Control and FGF1‐Tek mice were fasted overnight and administered a 1 g/kg dose of glucose by I.P. injection. Serum glucose levels were measured using the OneTouch Ultra Glucometer (LifeScan, Inc.) at 0, 20, 40, 60, and 120 min post injection.

### Serum insulin ELISA

Female FVB Control and FGF1‐Tek mice subjected to the 4‐week high‐fat diet protocol were fasted 6 h prior to administration of a 1 g/kg dose of glucose by I.P. injection. Mice were euthanized 30 min after the glucose bolus and whole blood was collected by heart puncture. Serum was separated from blood cells by centrifugation and stored at −79°C until use. Serum insulin levels were determined for each sample in duplicate per manufacturer's instructions (Calbiotech, Insulin Elisa kit # IS130D). Concentration of serum insulin was determined for each sample in duplicate using a standard curve as described within the instructions.

### Serum adiponectin, leptin ELISA assays

Female FVB Control and FGF1‐Tek mice subjected to the 4‐week high‐fat diet protocol were fasted 6 h prior to euthanasia and whole blood was collected by heart puncture. Serum was separated from blood cells by centrifugation and stored at −79°C until use. Adiponectin concentration was analyzed following manufacturer's instructions using a 1:200 dilution of serum in the product's reagent diluent (R&D Systems, Inc. Murine Adiponectin Duo kit #DY1119). Serum adiponectin concentration was determined for each sample in duplicate using a standard curve as recommended by the manufacturer. Leptin concentration was analyzed following manufacturer's instructions using a 1:2 dilution of serum in the product's reagent diluent without Tween‐20 (R&D Systems, Inc. Murine Leptin Duo kit #DY 498). Serum leptin concentration was determined for each sample in duplicate using a standard curve as recommended by the manufacturer.

### Serum nonesterified fatty acid (NEFA) assays

Female mice subjected to the 4‐week high‐fat diet protocol were fasted 6 h prior to euthanasia and whole blood was collected by heart puncture. Serum was separated from blood cells by centrifugation and stored at −79°C until use. Free fatty acids were analyzed using the NEFA‐HR(2) kit per manufacturer's instructions (Wako Diagnostics). Serum NEFA concentration was determined for each sample in duplicate using a standard curve as recommended by the manufacturer.

### Histological analysis of visceral adipose and liver samples

Visceral adipose and liver samples were isolated after euthanasia, rinsed in phosphate buffered saline (PBS) and fixed 48–72 h in neutral formalin with rotation. Samples were then rinsed in PBS and stored in 70% ethanol. After embedding in paraffin, samples were sectioned at either 8 or 5 microns for adipose and liver, respectively. Sections were stained with either hematoxylin and eosin (H&E; liver and adipose) or Masson's trichrome (adipose, only). Images of cell sections were obtained using bright field microscopy at 100X and 400X using a Nikon Eclipse E400 microscope equipped with a Lumenera Infinity 3.3 MP color CCD camera (Ottawa, Ontario) at magnifications shown within the figures. The Image J program (http://imagej.nih.gov/ij/) was used to determine average cell size/area. “Beige fat” area was determined by using Image J to analyze pixels corresponding to beige fat area/total area of the entire fat pad section on pictographs generated by scanning Masson's trichrome‐stained sections with the LI‐COR Odyssey Infrared System set to 680 nm.

### Quantitative reverse‐transcriptase polymerase chain reaction (qRT‐PCR) assays

Total RNA was isolated from visceral adipose using Tri‐Reagent (Sigma, Inc). cDNA was generated from 0.5 *μ*g of RNA using Iscript gDNA Clear cDNA Synthesis kit (Biorad, Inc.) which removes any genomic DNA prior to cDNA synthesis. Taqman‐based qRT‐PCR was performed on all samples in triplicate using Taqman‐Fast reagents and Taqman Assays on an ABI Step‐One instrument. Relative expression was determined using the ΔΔC_T_ method. Taqman assays used for these studies are as follows: murine PPAR*γ* (Mm00440940_m1); murine UCP1 (Mm01244861_m1) and murine PRDM16 (Mm00712556_m1).

### Cell cultures

Primary stromal‐vascular cells from visceral adipose were isolated and differentiated in 12 well tissue culture dishes as described (Hausman et al. 2008; Urs et al. 2012). Ear‐derived Mesenchymal Cell Cultures (EMSC) were provided as a kind gift from Robert Koza, Ph.D. (MMCRI, Scarborough, Maine) and isolated and differentiated as described (Chu et al. 2014). Primary visceral SV and EMSC cultures were maintained in DMEM:F12 (50:50) media supplemented with 1% antibiotic/antimycotic (Gibco BRL) and differentiated with glucocorticoids, IBMX, and insulin as described (Urs et al. 2012). Differentiated cell cultures were fixed in neutral formalin and stained with Oil‐Red‐O. Oil‐Red‐O positive colonies were imaged and colonies counted/area using the “cell counting” feature of Image J. Three random areas/well (in a 12 well plate) were counted and averaged for each colony. Oil‐Red‐O uptake was measured by extracting the stained cultures with 90% isopropanol and reading the absorbance at 492 nm. Absorbance was normalized to cell number/plate.

### Statistics

For each measurement, two‐sample *t*‐test (with or without Welch's correction) was used to determine if the population means were equal for the FVB Control and the FGF1‐Tek groups. A 95% confidence interval was constructed for each measurement to estimate the difference in the population means for the two groups. For the energy expenditure measurements, analysis of covariance (ANCOVA) was used to determine if the population means are equal for the FVB Control and the FGF1‐Tek groups, after adjusting the effect of body mass on the energy expenditure measurements (Speakman 2013). For data that were not normally distributed, significance was determined using the Mann–Whitney U test for nonparametric data as indicated in figure legends.

## Results

**Figure 1:**
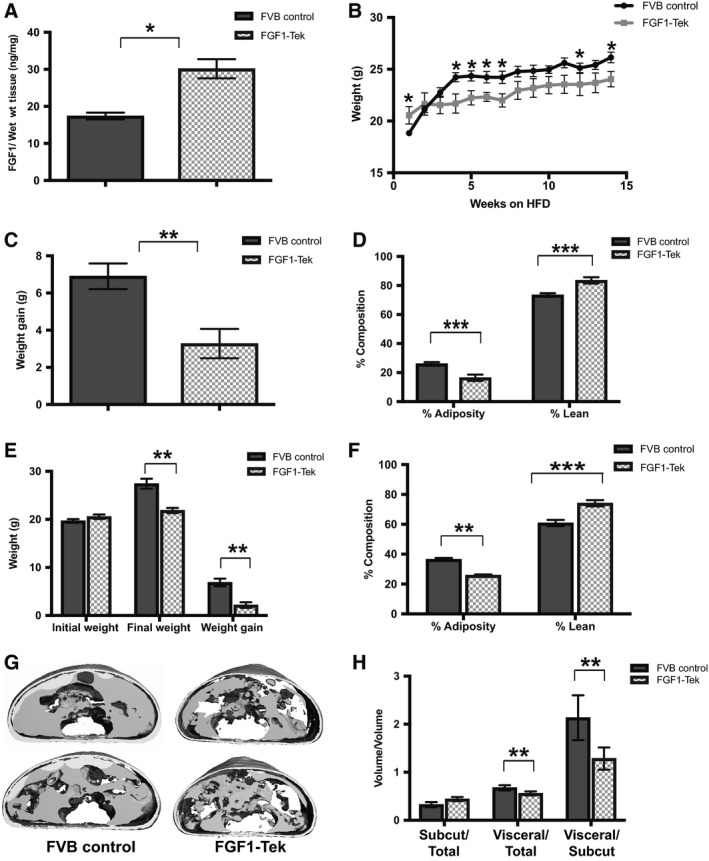
FGF1‐Tek mice gain less weight and are leaner than FVB Control animals after administration of a diet high in saturated fats. (A) FGF1 concentrations (ng/mg wet weight tissue) were determined by ELISA (R&D human FGF1‐Duo kit # DY232) in protein lysates obtained from visceral adipose of female mice placed on a normal chow diet and exposed to 0.66 *μ*g/mL doxycyline for 1 week. FGF1 concentration was significantly increased (*P* = 0.03) in the FGF1‐Tek mice (hatched bar) over FVB Control animals (solid bar) as determined by Student's *t*‐test with *n* = 3 and *n* = 5 for FVB Control and FGF1‐Tek cohorts, respectively. (B) Body mass (grams) of 5‐week‐old female FGF1‐Tek (*n* = 12) and FVB Control (*n* = 8) mice measured every week during administration of a 45% saturated fat diet over 14 weeks. Error bars represent SEM and significance was determined using Student's *t*‐test with significant differences in average body mass (“weight”) with *P* < 0.05 are indicated by *. (C) Average “weight” gain represents difference between the initial and final body mass of female FGF1‐Tek (hatched bars) and FVB Control (solid bar) mice in the study described under “B”. Error bars represent SEM and significance was determined using Student's *t*‐test with *P* = 0.0014. (D) Percent (%) adiposity and lean mass of female FVB Control (solid bar) and FGF1‐Tek (hatched bar) described under “B” was determined by PIXImus dual energy X‐ray absorptiometry. Data represent means for % adiposity and lean mass as indicated with error bars representing SEM and a significant difference of ***P < 0.001 determined by Student's *t*‐test. (E) Initial and final body mass (grams) as well as “weight” gain of 5‐week‐old female FGF1‐Tek (hatched bars, *n* = 12) and FVB Control mice (solid bars, *n* = 11) measured in newly‐weaned female FVB WT control (solid bars) and FGF1‐Tek (hatched bars) after administration of a 45% saturated fat diet over 4 weeks. Error bars represent SEM and significance was determined using Student's *t*‐test with significant differences in final body mass and “weight” gain at **P < 0.01. (F) Percent (%) adiposity and lean mass of female FVB Control (solid bar) and FGF1‐Tek (hatched bar) described under “E” as determined by PIXImus dual energy X‐ray absorptiometry. Data represent means for % adiposity or lean mass with error bars representing SEM and a significant difference of **P < 0.01 or ***P < 0.001 as determined by Student's *t*‐test as indicated. (G) Representative microtomography (*μ* CT) images obtained after scanning the torso of FVB Control or FGF1‐Tek female mice described under “E”. Solid material surrounding the interior central core of the body cavity represents omental adipose. (H) Adipose volumes and density were determined from microtomography (*μ* CT) images obtained from FVB Control (solid bar) and FGF1‐Tek (hatched bar) mice described under “E” and are reported as a ratio of fat type to total adipose volume (first and second panels) or as a ratio of visceral volume/subcutaneous volume as indicated. Significance was determined using Student's *t*‐test with *P* values **<0.01 for both visceral/total adipose volume and visceral/subcutaneous ratios. Subcutaneous/total volume was not significantly different between the two strains.

FGF1‐Tek mice express an endothelial‐cell specific, doxycycline‐inducible FGF1 transgene that results in the secretion of FGF1 into all vascularized tissues including adipose (Kirov et al. 2012). FGF1 is released from endothelial and other cells through the mechanisms of nonclassical secretion and its secretion is enhanced under conditions of hypoxia as well as conditions of thermal and oxidative stress (Prudovsky et al. 2002, 2016; Duarte et al. 2008; Kirov et al. 2015). Since the FGF1‐Tek strain was backcrossed to the FVB/N strain, these animals were used as wild‐type controls (FVB Control) (Kirov et al. 2012). Although the C57Bl/6J is the predominant strain used in diet‐induced obesity and insulin resistance studies (Berglund et al. 2008; Kim et al. 2013), we and others (Nascimento‐Sales et al. 2017) have found that the FVB/N strain does indeed gain visceral adipose and exhibits characteristics of metabolic syndrome when administered a diet high in saturated fats. An FGF1 ELISA on visceral adipose tissue protein extracts from both FVB Control and FGF1‐Tek mice confirmed that FGF1 was elevated in visceral adipose in our model (Fig. 1A). The measured concentration of FGF1 per mg wet weight tissue in the FGF1‐Tek mouse was approximately twofold higher as compared to FVB Control animals, indicating that transgenic FGF1 produced in endothelial cells was subsequently secreted into the visceral adipose depot.

### Transgenic expression of FGF1 suppresses visceral adipose accretion

To test our hypothesis that FGF1 reduces the impact that a diet high in saturated fats (HFD) has on adiposity, we measured weight gain and body composition of female FVB control and FGF1‐Tek mice administered a 45% saturated fat diet for 14 weeks starting at 5 weeks of age (Fig. 1B–D). Although FGF1‐Tek mice weighed slightly more than their FVB control counterparts at the start of the study, weight gain in the FGF1‐Tek mice was twofold less than FVB controls after 14 weeks on the HFD (Fig. 1B and C). In the 14‐week study, the rate of weight gain in FVB controls rose sharply during the first 3 weeks of HFD before plateauing (Fig. 1B). In contrast, weight gain in FGF1‐Tek mice was marked by a slower and less dramatic increase after the first week of the study (Fig. 1B). Importantly, FGF1‐Tek mice were leaner with significantly less abdominal fat accretion as compared to FVB controls (Fig. 1D). Similar patterns of weight gain and adiposity were also found in female mice administered the HFD for 4 weeks starting at 5 weeks of age (Fig. 1E and F). Interestingly, in vivo tomography confirmed a marked decrease in visceral adipose volume in the FGF1‐Tek cohort, although the subcutaneous volume was not significantly changed by transgenic FGF1 expression (Fig. 1G and H). Thus, the ratio of visceral/subcutaneous adipose – a measure that correlates with increased incidences of cardiovascular and metabolic disease (Tai et al. 2000; Wajchenberg et al. 2002; Porter et al. 2009; Kaess et al. 2012) – was over 1.5‐fold higher in FVB Control animals compared to FGF1‐Tek mice (Fig. 1H). Although average weight gain was also decreased in FGF1‐Tek males administered a HFD, aggression among littermates housed in the same cage introduced another variable that impacted this measure (data not shown). Together, these data support a protective role of FGF1 against visceral adipose accumulation, even under conditions where intake of saturated fat is excessive over an extended period.

### Transgenic expression of FGF1 increases physical activity and food consumption

**Figure 2:**
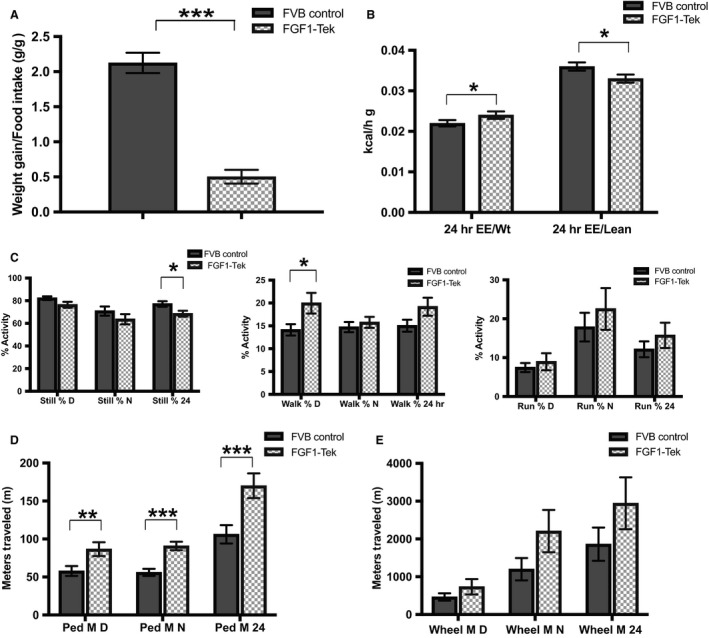
FGF1‐Tek mice display lower resting metabolic rates but are significantly more active than FVB Control strains. Five‐week‐old female FVB Control (solid bar, *n* = 13) and FGF1‐Tek (hatched bar, *n* = 12) mice were administered a 45% saturated fat diet for 3 weeks and then placed in Promethian Metabolic Chambers for 5 days during the fourth week of the study. Continuous measurements of mouse body mass (“weight”), locomotor activity, oxygen consumption, CO_2_ production and food intake were performed. Ratios of weight gain/food intake(A), energy expenditure/weight or lean mass (B), % time still, walking or running while awake (C), total meters traveled (D), and total meters traveled on the wheel (E) are reported as indicated. Statistical significance was determined using two‐tailed, unpaired Student's *t*‐test and error bars represent SEM with *P < 0.05, **P < 0.01; ***P < 0.001 as indicated.

**Figure 3:**
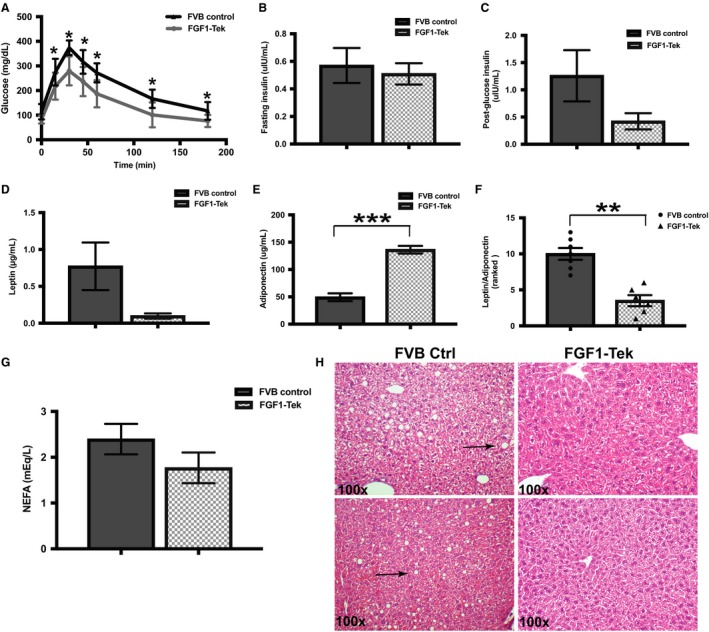
Markers of glucose tolerance and lipid metabolism are improved in FGF1‐Tek mice compared to FVB Controls after administration of a diet high in saturated fats. (A) Young, female FVB Control (circles, *n* = 9) or FGF1‐Tek mice (squares, *n* = 10) were placed on a 45% saturated fat diet for 4 weeks and fasted for 6 h before subjected to the glucose tolerance test as described under Materials and Methods. Data points were significant for each time point (*P* values from 0.03 to 0.0005) as well as for total area under the curve (*P* = 0.0003) as determined by Student's *t*‐test with Welch correction. (B) Mean insulin concentration in FVB Control mice (solid bars, *n* = 7) and FGF1‐Tek (hatched bars, *n* = 7) administered a 45% saturated fat diet for 4 weeks and fasted for 6 h before serum was obtained by heart puncture at the time of euthanasia. Error bars represent SEM and significance was determined using Student's *t*‐test. (C) Mean serum insulin concentration in FVB Control mice (solid bars, *n* = 4) and FGF1‐Tek (hatched bars, *n* = 4) administered a 45% saturated fat diet for 4 weeks and fasted for 6 h before administration of a 1 g/kg body weight dose of glucose by IP injection. Serum was obtained by heart puncture at the time of euthanasia thirty minutes after glucose administration. Error bars represent SEM and significance was determined using Student's *t*‐test with Welch correction. (D) Mean serum leptin concentration in FVB Control mice (solid bars, *n* = 8) and FGF1‐Tek (hatched bars, *n* = 8) administered a 45% saturated fat diet for 4 weeks and fasted for 6 h before serum was obtained by heart puncture at the time of euthanasia. Error bars represent SEM and significance was determined using Student's *t*‐test with Welch correction. (E) Mean serum adiponectin concentration in serum obtained from mice described in “D”. Error bars represent SEM and significance was determined using Student's *t*‐test with Welch correction. (F) Ranked means of ratios of serum leptin/adiponectin concentrations of mice used in “D” and “E”. Significance (*P* < 0.01) was determined using Mann‐Whitney test for nonparametric data. (G) Mean serum nonesterified fatty acids from female FVB Control (solid bars, *n* = 4) and FGF1‐Tek (*n* = 6) mice of mice described in “D”. Error bars represent SEM and significance was determined using Student's *t*‐test with Welch correction (*P* = 0.0613). (H) Representative H&E ‐stained sections of livers taken from FVB Control and FGF1‐Tek mice described under “D” are shown. Images are at 100 × magnification from two different animals of each strain. Morphological structures shown as small, round clear spots in tissue (arrows) indicate evidence of lipid droplets before tissue fixation.

In order to determine the mechanism(s) underlying FGF1‐mediated resistance to visceral adipose accretion, we obtained a metabolic profile of FVB Control and FGF1‐Tek mice using an established metabolic chamber assay (Demambro et al. 2012). We limited these studies to females because the difference in weight gain induced by a diet enriched in saturated fats was most pronounced in this sex and as previously indicated, male FGF1‐Tek were prone to aggressive behavior that impacted the study. Continuous measurements of mouse weight, exercise frequency, oxygen consumption, CO_2_ production, food and water intake as well as locomotor activity were obtained from young female FVB Control and FGF1‐Tek mice administered the diet high in saturated fats for 3 weeks previous to being individually placed into Metabolic Chambers. The HFD was continued while the mice were kept in the chambers for 5 days. Surprisingly, daily chow intake was 14% higher in FGF1‐Tek mice than in FVB Control animals (Table 1), indicating that the increase in transgenic FGF1 was not reducing food intake as had been reported in studies where rodents were administered recombinant FGF1 through injection (Suh et al. 2014). These results may be explained by both a marked decrease in serum leptin (Fig. 3D) as well as an unknown effect of FGF1 on appetite centers in the hypothalamus. Differences in weight gain were also not attributable to changes in metabolic processing of fats and carbohydrates as the respiratory quotients for both lines were not significantly different (Table 1). However, weight gain per gram of food consumed was fivefold lower in FGF1‐Tek mice (Fig. 2A), suggesting that FGF1 was increasing caloric expenditure either by raising basal metabolic rates and/or increasing physical activity. To determine if either of these factors were contributing to the resistance to weight gain observed in the FGF1‐Tek strain, we normalized total energy expenditure to weight gain and found this ratio was significantly increased in FGF1‐Tek mice as compared to FVB Controls (Fig. 2B, Table 1). Surprisingly, when normalized to lean mass, energy expenditure was actually lower in the FGF1‐Tek strain (Fig. 2B, Table 1). Furthermore, when total energy expenditure was delineated into resting and active metabolic rates and subject to Analysis of Covariance after removing the effect of body weight (Speakman 2013), the mean resting metabolic rate was also found to be significantly lower in the FGF1‐Tek cohort (*P*‐value = 0.02198). Therefore, constitutive endothelial expression of FGF1 appears to decrease, not increase, basal metabolism in our model.

**Table 1:** Metabolic measurements of FGF1‐Tek and FVB Control mice administered a HFD for 4 weeks

Measurement	FVB Control (mean ± SEM)	FGF1‐Tek (mean ± SEM)	Mean difference (FGF1Tek‐ FVB)	95% CI (lower limit)	95% CI (upper limit)	*P* Value
24 h energy expenditure (Kcal/h)	0.574 ± 0.011	0.539 ± 0.006	−0.035	−0.061	−0.009	**0.010**
24 h resting energy expenditure (kcal/h)	0.554 ± 0.01	0.495 ± 0.008	−0.058	−0.086	−0.031	**0.0001**
24 h active energy expenditure (kcal/h)	0.671 ± 0.004	0.651 ± 0.011	−0.019	−0.045	0.006	0.183
24 h energy expenditure/weight	0.022 ± 0.001	0.024 ± 0.001	0.002	0.0001	0.005	**0.039**
24 h energy expenditure/lean mass	0.036 ± 0.001	0.033 ± 0.001	−0.003	−0.004	−0.0004	**0.025**
Respiratory quotient 24 h	0.787 ± 0.009	0.797 ± 0.011	0.009	−0.021	0.04	0.537
Ped meters 24 h (m)	106.2 ± 12.063	170.15 ± 16.222	63.95	21.822	106.077	**0.005**
Ped speed 24 (m/h)	0.014 ± 0.001	0.015 ± 0.0003	0.001	−0.0002	0.002	0.095
24 h run %	12.15 ± 2.029	15.739 ± 3.249	3.59	−4.44	11.623	0.361
24 h walk %	15.035 ± 1.391	19.186 ± 1.964	4.15	−0.793	9.093	0.095
24 h still %	77.088 ± 2.469	68.497 ± 2.638	−8.591	−16.084	−1.097	**0.027**
24 h sleep	11.216±	9.979±	−1.237	−4.745	2.274	0.471
24 h X breaks	14,485 ± 1359	19,515 ± 2588	5031	−1147	11,208	0.104
24 h Y breaks	10,827 ± 845	17,267 ± 1194	6440	3388	9492	**0.0003**
24 h Z breaks	12,781 ± 941	11,457 ± 791	−1324	−3870	1222	0.293
24 h wheel distance (m)	1862.167 ± 440.032	2944.833 ± 686.552	1082.667	−625.771	2791.105	0.200
24 h average wheel speed	0.169 ± 0.013	0.181 ± 0.02	0.013	−.036	0.062	0.592
24 h food consumption	2.364 ± 0.101	2.746 ± 0.167	0.382	−0.027	0.791	**0.0654**
Weight gain/24 h food consumed (grams/grams)	2.125 ± 0.144	0.504 ± 0.098	−1.622	−1.99	−1.253	**<0.0001**
Water intake	1.005 ± 0.105	1.607 ± 0.169	0.602	0.185	1.019	**0.007**

Bolded numbers highlight p‐values for measures found to be significantly different between FVB Control and FGF1‐Tek animals.

Since reduction in adipose could also be caused by an increase in physical activity, both strains of mice were analyzed for movement type (walking, running, wheel usage), distance traveled and speed of movement. Sleep periods were similar between the FVB Control and FGF1‐Tek strains (Table 1); however, the FGF1‐Tek mice moved more often while awake than FVB Control animals (Fig. 2C, Table 1) and both distance traveled (Fig. 2D) and walking speed (Table 1) were significantly increased in FGF1‐Tek mice. FGF1‐Tek mice wheel usage was ~1.5‐fold higher than FVB Control mice (Fig. 2E, Table 1), although this difference was not significant due to high variability among individuals. Interestingly, the FGF1‐Tek mice did not increase all movements as neither standing on hind legs (z movements) nor movement across the long end of the cage (X breaks) were significantly different (Table 1). Instead, FGF1‐Tek mice preferred to run along the short side of the cage (Y Breaks, Table 1) – behavior that may be correlated with an increased occurrence of circling movements among these animals (data not shown) that could possibly be due to an unknown neurological effect of transgenic FGF1 overexpression. Together, these data suggest that excess FGF1 causes an increase in activity that may counter caloric intake.

### Transgenic expression of FGF1 improves glucose sensitivity, lowers serum‐free fatty acids and prevents diet‐induced liver steatosis

FGF1‐Tek mice consumed an abnormally large volume of water that was ~62% higher than that consumed by FVB Controls (Table 1). Since excessive thirst can be indicative of hyperglycemia and lipodystrophic syndromes are associated with diabetes (Joffe et al. 2001), we performed a glucose tolerance test. In agreement with published accounts supporting a positive role for FGF1 in maintaining glucose homeostasis (Jonker et al. 2012; Suh et al. 2014; Perry et al. 2015), we found that transgenic FGF1 significantly lowered glucose levels in the FGF1‐Tek mice when compared with FVB controls after fasting (*P* = 0.0003 for area under the curve, Fig. 3A). In addition, serum glucose was significantly lower at all time points after administration of the bolus of glucose in the FGF1‐Tek cohort (Fig. 3A). Fasting serum insulin was nearly identical for both FVB Control and FGF1‐Tek strains (Fig. 3B), but was threefold lower in the FGF1‐Tek animals after intraperitoneal administration of a glucose bolus (Fig. 3C). Since fasting serum concentrations and ratios of the adipokines adiponectin and leptin are often used as indicators of glycemic and adipose heath (Arita et al. 1999; Miller et al. 2011; Borges et al. 2018), we examined these hormones in our FVB Control and FGF1‐Tek animals administered a HFD for (4) weeks. Leptin was substantially higher (>10‐fold) in FVB Control animals than in FGF1‐Tek strains, although was not found to be statistically different due to the high variability in the control animals (Fig. 3D). Conversely, despite a marked decrease in white adipose in the FGF1‐Tek animals, the amount of adiponectin produced was nearly threefold higher compared to FVB Controls (Fig. 3E). An increased ratio of serum leptin/adiponectin has been correlated with metabolic syndrome and type II diabetes (Mojiminiyi et al. 2007; Finucane et al. 2009). In agreement with a glucose‐tolerant phenotype, the average leptin/adiponectin ratio in FGF1‐Tek mice is less than 50% of that measured in FVB Control animals (Fig. 3F). Together, these data indicate that overexpression of endothelial FGF1 not only reduces adiposity, but also confers protection against HFD‐induced insulin resistance.

Consumption of saturated fatty acids increases serum nonesterified fatty acids (NEFA) that can contribute to the development of liver steatosis (Lee et al. 2015; Liu et al. 2016a). Analysis of serum NEFA from fasted animals trended lower in FGF1‐Tek mice compared to FVB Control animals (Fig. 3F). Histological analysis of liver sections of FVB Control, but not FGF1‐Tek mice displayed morphological evidence of inappropriate lipid deposition resulting from the administration of the HFD (Fig. 3H). Visual and tactile analysis of the feces from FGF1‐Tek mice did not indicate that FGF1‐Tek mice failed to absorb saturated fats from the diet. Instead, these data suggest that dietary fatty acids are utilized more efficiently in the FGF1‐Tek mice, preventing harmful accumulation of NEFA in serum and liver.

### Transgenic expression of FGF1 impacts the cellular composition of visceral adipose

**Figure 4:**
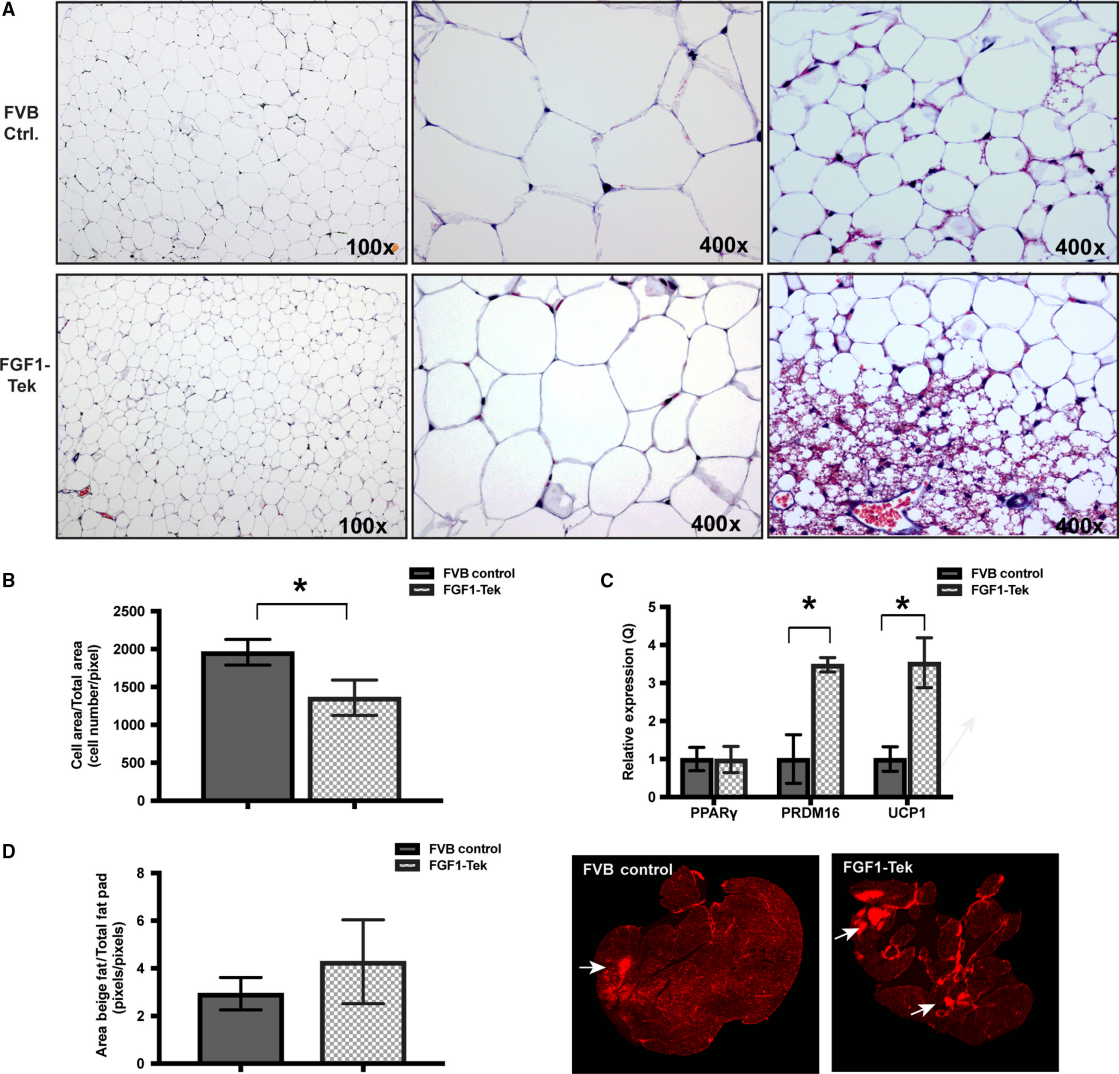
Visceral adipose morphology and cellularity differ between FVB Control and FGF1‐Tek strains. All analyses were performed on adipose obtained from the abdominal cavity of young, female FVB WT control and FGF1‐Tek mice administered HFD for 14 weeks as described under Figure 1B and in Materials and Methods. (A) Adipose removed from the abdominal cavity of FVB WT control (top row) and FGF1‐Tek mice (bottom row) were fixed in neutral formalin and stained with Masson's trichrome. Images are at either 100 × or 400 × magnification as shown. The last column represents 400 × magnification of regions containing what is morphologically consistent with “beige” fat. B. The average area of adipocytes was determined by counting the number of cells/section area (pixels) using Image J software. Three (3) random fields/fat pad were imaged for FVB WT controls (solid bars, *n* = 5) and FGF1‐Tek (hatched bars, *n* = 6). Error bars represent SEM and a significant difference of *P < 0.05 as determined by Student's *t*‐test with Welch correction as indicated. (C) Total RNA extracted from visceral adipose of FVB WT control (solid bars, *n* = 8) and FGF1‐Tek (hatched bars, *n* = 5) was reverse‐transcribed into cDNA and subject to quantitative RT‐PCR using Taqman™ Fast reagents and assays for murine PPAR *γ*, PRDM16 and UCP‐1 as indicated. Relative expression was determined using the ΔΔC_T_ method. (D) “Beige fat” area/fat pad was determined by imaging entire Masson's trichrome stained sections using the LiCor Odyssey infrared imager set to scan at 680 nm. Images adjacent to graph show that “beige fat” stained more intensely, producing a brighter dark red signal within the fat pad. Area occupied by “beige fat” was determined using Image J software to calculate the ratio of pixels in brightly stained regions to total area of the section. Error bars represent SEM and difference in staining intensity between the two strains as determined by Student's *t*‐test with Welch correction approached significance (*P* = 0.087).

To examine the effect that continuous FGF1 secretion had on visceral adipose morphology, we analyzed Masson's trichrome‐stained sections of adipose isolated from female FVB Control and FGF1‐Tek mice administered the HFD for 14 weeks (Fig. 4A). We determined that average adipocyte size was 1.85 larger in FVB WT controls than in FGF1‐Tek mice (Fig. 4B). Expression of mRNA encoding the master transcriptional factor PPAR*γ* in visceral adipose was not changed by transgenic FGF1 expression (Fig. 4C). Interestingly, visceral adipose pads of FGF1‐Tek mice displayed larger and more abundant areas within the depot that resembled “beige fat” marked by the presence of smaller, multilocular, and more densely clustered adipocytes that imparted a reddish‐purple tint in the trichrome‐stained tissue (Fig. 4A, bottom right panel; Fig. 4D). Visual evidence of “beiging” was supported by an increase in mRNA encoding the brown adipose factor PRDM16 (Kajimura et al. 2008; Cohen et al. 2014) and mitochondrial marker UCP1 (Fig. 4C). Thus, transgenic overexpression of endothelial FGF1 appears to impact the size and type of adipocytes residing within the visceral adipose of female mice exposed to a long‐term high‐fat diet.

**Figure 5:**
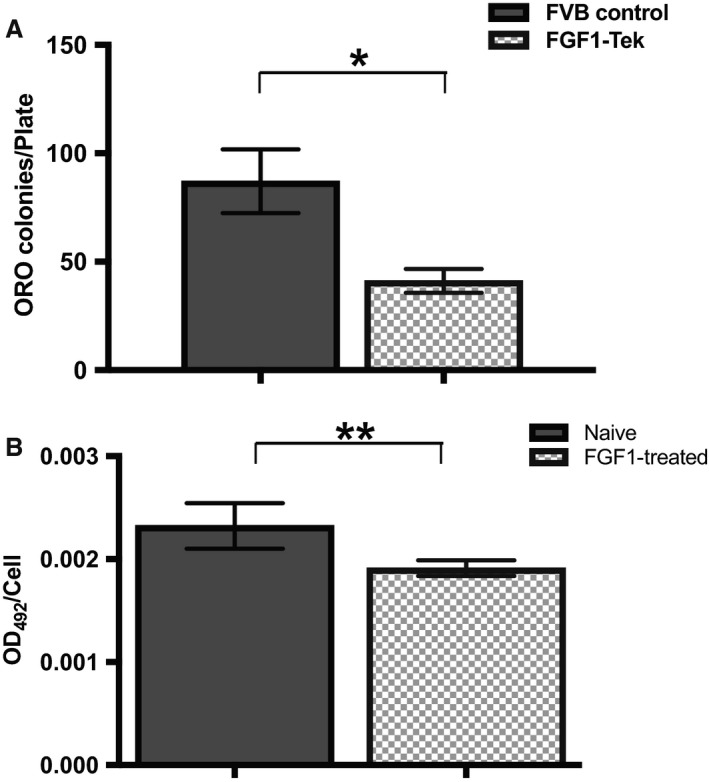
Continuous FGF1 stimulation attenuates adipogenesis in vitro. (A) The number of adipocyte colonies arising from primary stromal‐vascular cell cultures isolated from visceral adipose tissue of FVB Control (solid bars, *n* = 5) or FGF1‐Tek (hatched bars, *n* = 6) animals were determined 2 weeks after differentiation. The number of Oil‐Red‐O positive colonies captured on 100× micrographs were counted and normalized to culture area (pixels) using Image J software with 3 area/image averaged per well (total of 6 wells/culture). Error bars represent SEM of average colonies/culture and significance was determined using Student's *t*‐test (*P* = 0.0118). (B) Ear‐derived mesenchymal stem cells (EMSC) were incubated for 48 h with 0.2% serum (quiescence), followed by 36 h with 0.2% serum with (dark bar) or without (hatch bar) 10 ng/mL FGF1 prior to exposure to adipogenic differentiation media. Adipogenesis was measured by the extent of Oil Red O binding normalized to cell number. Error bars represent SEM of difference in staining intensity between the two strains as determined by Student's *t*‐test (*P* = 0.0016).

Although it is likely that the reduction in visceral adipose mass is due to a decreased need to store excess dietary fats in the FGF1‐Tek strain, it is also possible that prolonged exposure to FGF1 may be inhibiting preadipocyte proliferation and/or differentiation. To examine the effect of continuous FGF1 secretion into the white adipose depot, we differentiated primary stromal‐vascular cultures isolated from visceral adipose to determine if preadipocyte number and/or adipogenic potential differed between the FVB Control and FGF1‐Tek strains. Stromal‐vascular cell cultures from FGF1‐Tek mice produced 50% fewer Oil‐Red‐O positive colonies than FVB Controls (Fig. 5A), indicating that duration of FGF1 stimulation had a direct effect on adipogenic cell populations in white adipose. Interestingly, transient pretreatment with FGF1 also significantly reduced Oil‐Red‐O staining in differentiated primary EMSC cells (Fig. 5B). Together, these data suggest that repeated or long‐term FGF1 exposure may alter the ability of preadipocytes to replicate and/or differentiate.

## Discussion

We report that adolescent, female mice overexpressing an endothelial cell‐specific human *FGF1* transgene are resistant to visceral adipose accretion when administered a diet high in saturated fatty acids for up to 14 weeks. This is the first study, to our knowledge, to report the physiological effects of chronic, in vivo expression of transgenic FGF1 on weight gain, adiposity, whole body metabolism, visceral adipose accretion, and locomotor activity. Furthermore, we demonstrate that continuous FGF1 stimulation of preadipocytes impairs preadipocyte differentiation potential and induces “beiging” of the visceral depot. In our unique gain‐of‐function model, transgenic FGF1 is released continuously from vessels throughout the body; therefore, the observed phenotype likely reflects synergistic effects of systematic and prolonged FGF1 signaling not only in adipose, but in brain and other metabolic tissues including skeletal muscle and liver. For example, FGF1 regulates skeletal muscle development (Oliver et al. 1992; Conte et al. 2009) and its continuous expression, coupled with increased physical activity, may explain the significant increase in lean body mass observed in the FGF1‐Tek strain. Utilization of dietary fatty acids by skeletal and cardiac muscle in the leaner, more active FGF1‐Tek mouse would be expected to decrease the flux of lipids into the adipose depot, thus reducing the need for adipogenesis. A diminished requirement for lipid storage may also explain the smaller size of the FGF1‐Tek adipocytes within the visceral depot, precluding the harmful development of hypertrophic obesity in these animals. Finally, an increase in the presence of “beiged” fat cells that have characteristics of both white and brown adipocytes within the visceral adipose depot in the FGF1‐Tek mice may be an indirect effect of continuous exercise as physical activity has been shown to increase “beiging” (Cao et al. 2011; Bostrom et al. 2012; Stanford et al. 2015). This phenomenon is most often reported in the context of subcutaneous depots of both murine sexes; however, “beiging” of the visceral depot has been reported in response to inhibition of Follicle Stimulating Hormone (FSH) in female mice (Liu et al. 2017) and stimulation of Prostaglandin E2 (PGE2) (Garcia‐Alonso et al. 2016). Although FGF1‐Tek mice are fertile and display no abnormalities in litter size, it is possible that a combination of increased activity coupled with unknown effects of FGF1 on FSH or other sex hormones may promote an increase in patches of “beige” cells in the intraabdominal depot of our female mice.

Chronic release of FGF1 may ameliorate insulin‐desensitizing inflammatory signals generated by the over‐abundance of fatty acids in the diet. Our studies in the FGF1‐Tek mice agree with other in vivo studies that demonstrate FGF1 impacts insulin sensitivity and glucose tolerance (Jonker et al. 2012; Suh et al. 2014; Scarlett et al. 2016). Furthermore, serum leptin, adiponectin and the related measure of the leptin/adiponectin ratio were all dramatically affected by endothelial overexpression of FGF1. High leptin/adiponectin ratios have been correlated with the incidence of metabolic syndrome, cardiovascular disease, and type II diabetes (Mojiminiyi et al. 2007; Finucane et al. 2009; Titov 2014). Since low adiponectin levels are negatively associated with obesity, inflammation, decreased physical activity, and glucose intolerance (Yang et al. 2003; Kim et al. 2007; Mojiminiyi et al. 2007; Miller et al. 2011; Borges et al. 2018), it is likely that FGF1‐induced locomotor activity also enhances adiponectin production, which in turn may contribute to improved glucose homeostasis and insulin sensitivity in our model. Primary human preadipocytes exposed to FGF1 in cell culture systems express significantly more adiponectin and are more insulin‐sensitive then unexposed cells (Newell et al. 2006). Finally, increased adiponectin coupled with a faster utilization of the dietary fatty acids appears to prevent lipid accumulation in the livers of the FGF1‐Tek mice. Both FGF1 (Liu et al. 2016b) and adiponectin (Polyzos et al. 2011) have been shown to protect against nonalcoholic fatty liver disease (NAFLD), but whether or not FGF1 is acting through adiponectin or both work synergistically via different mechanisms to prevent this life‐threatening condition is not reported. Further analysis of the status of FGF‐signaling effectors may identify molecular mechanisms behind FGF1‐mediated effects on serum adiponectin levels as well as other metabolic targets that impact energy homeostasis and protect against organ damage caused by inflammation and dietary stress induced by a high saturated fat/high calorie diet.

It is of interest that while the metabolic and glucose tolerant phenotype of the FGF1‐Tek strain is similar to that observed in mice injected with rFGF1, there are notable behavioral differences. For example, in contrast the FGF1‐Tek mouse, acute administration of rFGF1 transiently decreased food intake (Scarlett et al. 2016). Furthermore, chronic administration of rFGF1 also did not increase physical activity (Suh et al. 2014), a phenotype that is, in contrast, evident in the FGF1‐Tek strain. Disruptions to glial and neuronal cell function resulting from HFD‐induced inflammation of the hypothalamus impair appetite and energy homeostasis (Thaler et al. 2012) and it is possible that continuous FGF1 release from the brain microvasculature exerts a protective effect against this stressor. Indeed, intracerebroventricular injection of rFGF1 has been reported to modulate hypothalamic activity (Matsumoto et al. 1998) and improve glucose and lipid metabolism (Perry et al. 2015) in rats. Interestingly, the FGF1‐Tek mouse is not only more active than controls, but also more aggressive and displays an abnormal behavioral phenotype (Small and Prudovsky, in preparation). These observations suggest that effects of FGF1 administration are complex and any therapies utilizing this potent cytokine must be approached cautiously to ensure that harmful behavioral side effects do not occur.

Reports on FGF1 effects on adipogenesis are varied and dependent on cell type and duration of exposure. For example, FGF1 was deemed essential for the survival and differentiation of cultured primary human preadipocytes by stimulating both clonal expansion and enhancing expression of PPAR*γ* (Hutley et al. 2004; Widberg et al. 2009). However, FGF1's ability to promote adipogenesis is also dependent on the timing and duration of FGF1 exposure and continuous administration of FGF1 can also suppress differentiation of human preadipocytes (Hutley et al. 2011). Indeed, we observed a reduction in the number of Oil‐Red‐O positive colonies from primary stromal‐vascular cultures obtained from FGF1‐Tek adipose, indicating that continuous FGF1 exposure may prevent the establishment of preadipocyte populations. This observation was substantiated by a similar effect on EMSC adipogenic differentiation. We have previously reported that repeated FGF1 stimulation also suppresses the proliferative response of endothelial cells and fibroblasts, a phenomenon we have coined as “FGF1 memory” (Poole et al. 2014, 2016). Together, these data strongly suggest that continuous or prolonged FGF1 exposure suppresses preadipocyte self‐renewal and/or the adipogenesis process itself. Thus, the lean phenotype observed in the FGF1 mouse is likely due to both an increase in energy utilization and a decrease in the ability to produce mature adipocytes.

Finally, although most diet‐related and metabolic studies are performed in the C57Bl/6J strain, our transgenic line was maintained in an FVB/N background. Although this strain has been reported to be resistant to diet‐induced obesity by other groups (Kim et al. 2013), we report here that these mice do indeed gain white adipose and show biochemical markers of insulin‐resistance. Our data in the FVB control animals are supported by a study by Nascimento‐Sales et al. 2017 that report diet‐induced weight gain, steatosis, and insulin‐resistance. Importantly, all significant differences in the physiological, cellular and biochemical responses of the FGF1‐Tek strain were compared to the FVB Control animals. These data also support and strengthen conclusions made by other groups using C57Bl/6J strains regarding the importance of FGF1 in ameliorating diet‐induced metabolic derangements.

In conclusion, our data suggest that FGF1 expression may be one genetic determinant behind an individual's tendency to resist obesity and/or metabolic disease even when consuming a diet high in saturated fats. Further exploration of mechanisms of FGF1 regulation of adipose development and metabolism in response to dietary stress may provide an experimental framework linking the incidence and treatment of obesity and related pathologies to the status of FGF1 gene expression in humans. Finally, although FGF1 administration may negatively impact behavior, our results show that it is a promising approach to the treatment of obesity and metabolic disease.

## Conflict of Interest

None.
